# A novel brief treatment for methamphetamine use disorders in South Africa: a randomised feasibility trial

**DOI:** 10.1186/s13722-020-00209-3

**Published:** 2021-01-07

**Authors:** K. Sorsdahl, D. J. Stein, S. Pasche, Y. Jacobs, R. Kader, B. Odlaug, S. Richter, B. Myers, J. E. Grant

**Affiliations:** 1 Alan J. Flisher Centre for Public Mental Health, Department of Psychiatry & Mental Health, University of Cape Town, 46 Sawkins Rd., Cape Town, 7700 South Africa; 2 Department of Psychiatry & Mental Health, University of Cape Town, Cape Town, South Africa; 3 South African Medical Research Council Unit on Risk & Resilience in Mental Disorders, Cape Town, South Africa; 4 Department of Psychology, Stellenbosch University, Stellenbosch, South Africa; 5 Alcohol, Tobacco and Other Drug Research Unit, South African Medical Research Council, Cape Town, South Africa; 6 Clinical and Translational Science Institute, University of Minnesota, Minneapolis, MN USA; 7 Professional Data Analysts, Minneapolis, United States; 8 Department of Psychiatry & Behavioural Neuroscience, University of Chicago, Chicago, United States

**Keywords:** Feasibility trial, Substance use, Brief treatment, South Africa

## Abstract

**Background:**

Effective brief treatments for methamphetamine use disorders (MAUD) are urgently needed to complement longer more intensive treatments in low and middle income countries, including South Africa. To address this gap, the purpose of this randomised feasibility trial was to determine the feasibility of delivering a six-session blended imaginal desensitisation, plus motivational interviewing (IDMI) intervention for adults with a MAUD.

**Methods:**

We enrolled 60 adults with a MAUD and randomly assigned them 1:1 to the IDMI intervention delivered by clinical psychologists and a control group who we referred to usual care. Feasibility measures, such as rates of recruitment, consent to participate in the trial and retention, were calculated. Follow-up interviews were conducted at 6 weeks and 3 months post-enrollment.

**Results:**

Over 9 months, 278 potential particiants initiated contact. Following initial screening 78 (28%) met inclusion criteria, and 60 (77%) were randomised. Thirteen of the 30 participants assigned to the treatment group completed the intervention. Both psychologists were highly adherent to the intervention, obtaining a fidelity rating of 91%. In total, 39 (65%) participants completed the 6-week follow-up and 40 (67%) completed the 3-month follow-up. The intervention shows potential effectiveness in the intention-to-treat analysis where frequency of methamphetamine use was significantly lower in the treatment than in the control group at both the 6 week and 3-month endpoints. No adverse outcomes were reported.

**Conclusions:**

This feasibility trial suggests that the locally adapted IDMI intervention is an acceptable and safe intervention as a brief treatment for MAUD in South Africa. Modifications to the study design should be considered in a fully powered, definitive controlled trial to assess this potentially effective intervention.

*Trial registration* The trial is registered with the Pan African Clinical Trials Registry (Trial ID: PACTR201310000589295)

## Background

Substance use disorders represent a major public health problem, both globally and in South Africa. Substance use contributes significantly to the global burden of disease, accounting for 11% of total health burden [1]. Similar to other low and middle income countries, data from a nationally representative community sample in South Africa indicate a high lifetime prevalence (13.3%) and early onset (21 years) of substance use disorders [2]. In the Western Cape Province, the primary substance of abuse is alcohol, but recent years have seen increases in the use of methamphetamine (MA) [3] which is now the third most commonly used substance (after alcohol and cannabis) among people seeking substance use treatment in the Western Cape. Although nationally representative prevalence data on MA use does not exist, a study investigating drug and human immunodeficiency virus (HIV) risk behaviour in 1379 South Africans from three communities reported that 7.3% of those interviewed had used MA more than once [4].

MA use is associated with several negative sequelae. These include a range of psychiatric symptoms such as depression and suicide, as well as serious medical conditions such as cardiac arrhythmias and myocardial infarction [5–8]. In addition, MA use has been associated with neurocognitive impairment, interpersonal violence and risk of HIV acquisition and transmission [9, 10], two key drivers of South Africa’s burden of disease.

Given the psychological and public health implications associated with MA use, researchers from high-income countries have investigated the efficacy of a variety of psychological treatments for MA use [11]. Contingency management, interpersonal therapy, cognitive behavioural therapy (CBT) (including the Matrix Model), and motivational approaches, such as motivational interviewing, have shown to reduce MA use [11–14]. Despite the availability of effective treatments, many people globally with a methamphetamine use disorder (MAUD), do not receive adequate treatment [15].

This is also true for South Africa, where community-based studies have found that although people who use MA would generally meet diagnostic criteria for a severe substance use disorder and recognise the need to get treatment [16, 17], less than 3% of MA users who need treatment ever access these services [16, 17] due to a variety of structural barriers [18, 19]. A particularly salient barrier is the limited allocation of funding resulting in many non-for profit facilities being unable to continue to provide free services to clients from vulnerable communities [16]. Further, the existing substance abuse treatment system in South Africa relies heavily on the provision of high threshold, drug “rehabilitation” services offered by specialised service providers. For example, in the Western Cape Province, the Matrix Model is offered at various outpatient services and consists of a 16-week treatment programme that incorporates elements from motivational interviewing, cognitive behavioural treatment and contingency management through individual, group, and family sessions [20, 21]. While this is a programme of known efficacy, the programme’s coverage is not sufficient to meet population needs for this service, particularly among disadvantaged communities [18]. In addition, the programme is very long and attrition rates are high [22]. To address this treatment gap and respond to the MA epidemic, briefer efficacious MA treatments are needed to complement the high threshold services that are available.

One brief treatment with potential for use in South Africa to treat MAUD is a manualized programme that includes components of cognitive-behavioural therapy, motivational interviewing, and imaginal exposure developed by Grant et al. [23]. Imaginal desensitisation plus motivational interviewing (IDMI) is a structured, six-session brief treatment with evidence of effectiveness for the treatment of impulse control disorders in the United States [24], and has been adapted for the treatment of pathological gambling in South Africa [25]. This treatment approach may be helpful for MAUD given that many of the mechanisms underlying behavioural addictions (such as pathological gambling) and substance use disorders are shared [26]. However, unanswered questions about the feasibility of implementing this treatment, it’s acceptability and potential efficacy for addressing MAUD among South African populations needed to be addressed before recommendations about its use can be made.

### Trial objectives

The primary objectives of this study were to (i) examine the feasibility of a six-session brief IDMI treatment for South Africans with MAUD as well as; (ii) the feasibility of study procedures including the performance of primary and secondary outcome measures to inform a fully powered trial. A second objective was to explore the initial effect of this IDMI intervention on MA use.

## Methods

This paper has been written in accordance with the Consolidated Standards of Reporting Trials (CONSORT) 2010 statement: extension to randomised pilot and feasibility trials [27] and the Standard Protocol Items: Recommendations for Interventional Trials (SPIRIT) checklist [28].

### Participants

Sixty participants were recruited through several avenues including referral from health care services, local non-government organisations (NGOs) and advertisements in local newspapers. Eligible participants were between 18 and 65 years of age; had at least a grade 9 education (a proxy for English literacy), met Diagnostic and Statistical Manual fourth edition (DSM-IV) criteria for MA abuse or dependence according to the Mini-International Psychiatric Interview (MINI) substance use module, and self-reported MA as their drug of choice. Patients with other co-occurring substance use disorders were not excluded. Exclusion criteria included participants meeting criteria for bipolar disorder, suicidality, schizophrenia (including psychosis) and antisocial personality disorder (as assessed through the MINI).

### Procedure

**Fig. 1:**
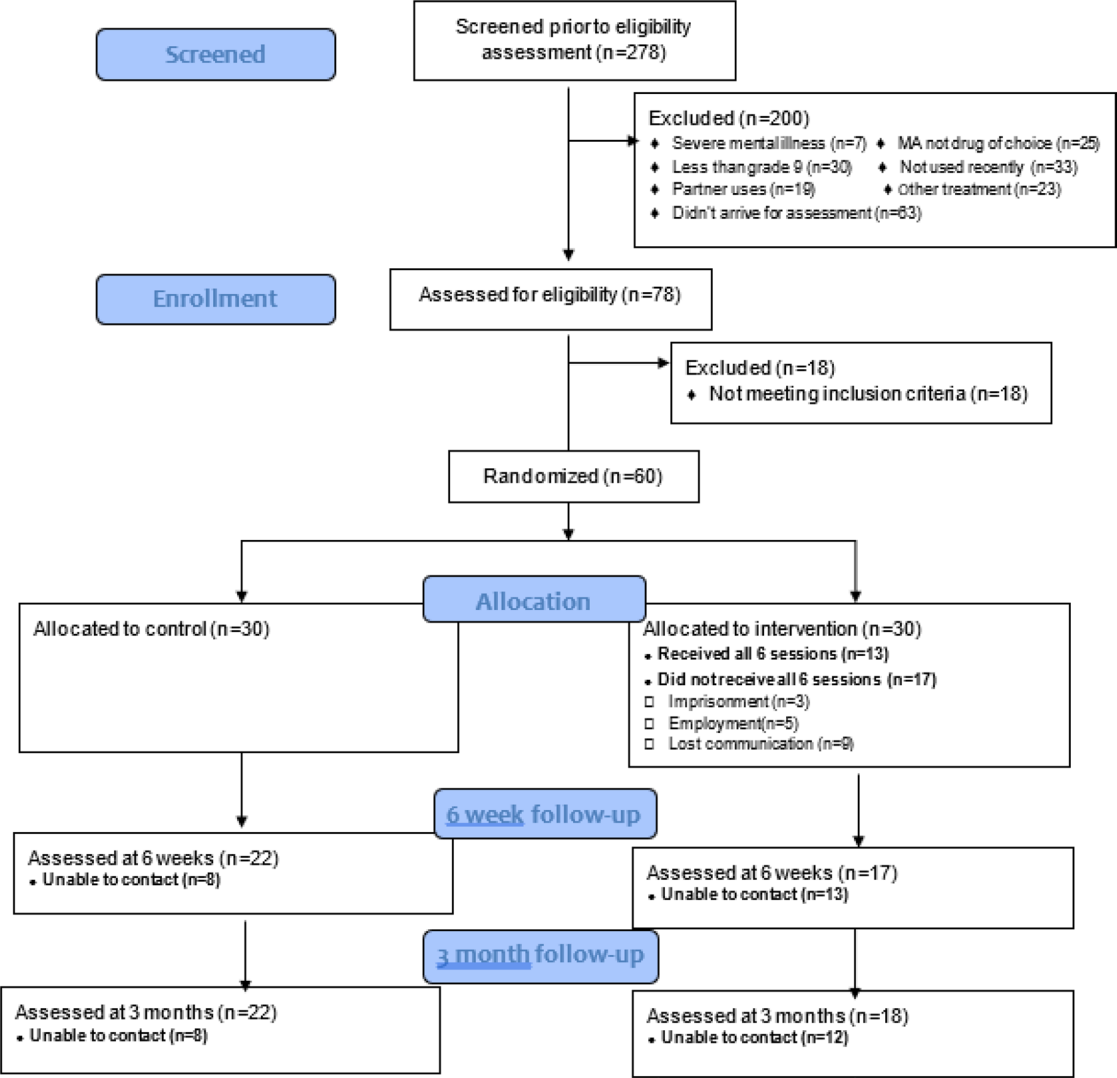
Consort diagram for feasibility study

See Fig. 1 for consort diagram. Interested participants contacted the study’s research assistant by phone or text message. A pre-screener was then administered over the phone. Questions pertaining to the age of the participant and frequency of drug use were elicited. All those not excluded from the pre-screener were asked to attend a screening assessment at Groote Schuur Hospital in Cape Town. During this assessment, all participants received a psychiatric assessment by a Registered Counsellor, with a 4-year honours degree in Psychology. Psychiatric diagnoses were obtained using the MINI. All participants that were excluded due to a dual diagnosis (bipolar disorder, schizophrenia and antisocial personality disorder) were referred to a dual diagnosis clinic located at the hospital for further assessment and treatment.

All participants who met criteria and consented to participate in the study completed an interviewer-administered baseline questionnaire on self-reported substance use and mental and physical health. They were then randomly assigned to the IDMI treatment or control group (1:1). The treatment group received the six-session treatment, while the control group were referred to local substance use treatment centres for treatment.

Follow-up interviews were conducted at 6 weeks and 3 months following study enrolment, at which time the baseline questionnaire was re-administered. Grocery vouchers of R50 (approximately $5) were given to participants at each research assessment. To limit attrition, telephone and text message reminders were sent to participants. Participants were also compensated for any travel expenses.

### Randomisation and blinding

Participants were randomly allocated in a 1:1 ratio to either the intervention or comparison arm. The randomisation sequence was prepared by the data manager (using a computer programme) and allocation done by a trial manager. To limit a socially desirable response set, a research assistant who was not involved in the recruitment of the participants and is not working at the hospital conducted the follow-up assessment and was blinded to the treatment allocation.

### Interventions

#### Treatment arm

The IDMI sessions included:

Session 1: A condensed form of motivational enhancement therapy. Included aneEvaluation of why you want to quit using MA and monitoring your MA use. In this session, the client and the therapist worked together to explore the level of motivation to quit MA use and facilitated change talk by using elements from a motivational interviewing framework.

Session 2: Financial planning. In this session, the client and therapist worked together to identify financial concerns (debt for example) associated with MA use and explored strategies to help improve the client’s financial situation.

Session 3: Behavioural intervention, preparation for MA use triggers. In this session, strategies to resist against MA use triggers were developed for implementation.

Session 4: Exposure therapy (via guided imagery). In this session, the therapist spent approximately 20 minutes asking the client to visualize and describe a typical day of MA use and all the senses, thoughts, emotions, and behaviours involved in MA use. At the peak of the client’s urge during the exposure (imagery), consequences of actions are introduced as well as coping mechanisms to help deal with these urges. This imagery process is written as a script and audio recorded for the client to listen to at home.

Session 5: Impulsive beliefs: cognitive therapy. This session involved the therapist and client working together to identify any thinking errors or erroneous beliefs that the client had which perpetuated his/her MA use. The goal was to learn how to develop healthy beliefs related to MA use.

Session 6: Relapse prevention. This session involved a review of all the skills and information learned throughout this programme. It focused on preparing the client for future triggers that will occur and which tempt him/her to engage in MA use. We did not formally evaluate knowledge gained.

Session 7: Family involvement (optional). This session was optional and only completed at the client’s discretion. It involved a discussion with the client and his/her family and the best role(s) all parties could play in the rehabilitation of the client.

#### Control arm

Participants randomized to the control arm were referred to a specialised, registered outpatient rehabilitation centre that was most convenient for them to attend. All outpatient facilities provide a combination of motivational interviewing and cognitive behavioural-based counselling that incorporated both individual and group counselling sessions.

### Training and fidelity

Two clinical psychologists with more than 5 years’ experience delivering cognitive behavioural therapy were recruited and trained to deliver the intervention. The clinicians received 3 full days of training in the treatment. Debriefing also occurred with the principal investigator as needed. To ensure intervention fidelity, all sessions were audio recorded and a random sample of 15 sessions from each psychologist was checked for fidelity using an intervention checklist that focused on intervention content. Both psychologists were highly adherent to the intervention, obtaining a fidelity rating of 91%.

### Measures

**Fig. 2:**
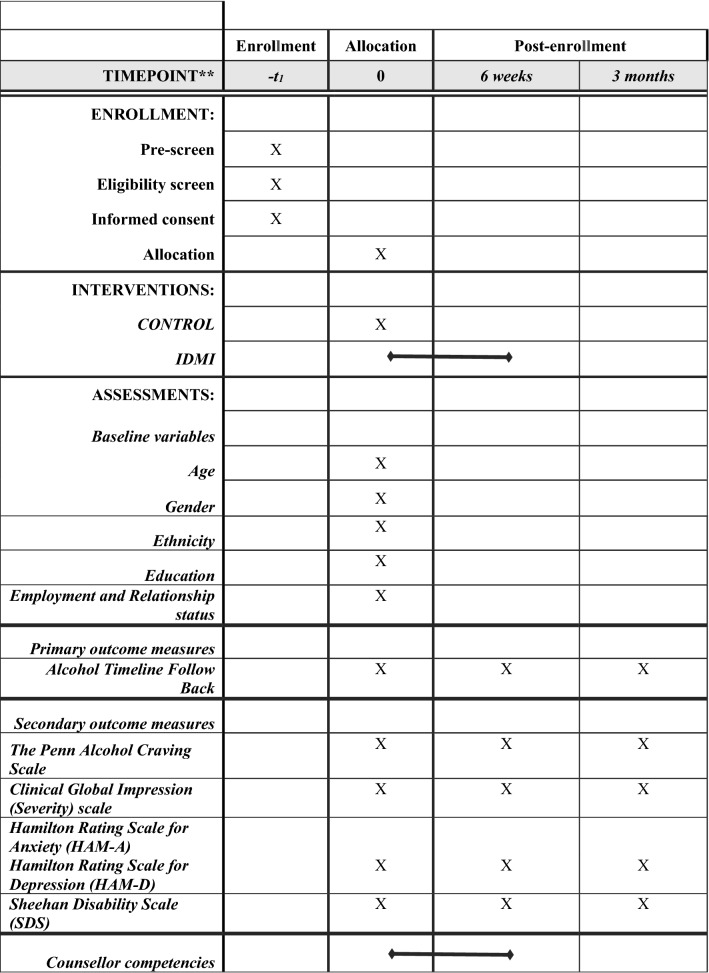
Standard Protocol Items: Recommendations for Clinical Trials (SPIRIT) Figure—schedule of data collection

The schedule of data collection is shown in Fig. 2 the SPIRIT Figure. Feasibility outcomes include: (1) feasibility of recruitment (numbers screened, number of eligible participants, number invited to participate, consent rates (for parents and adolescents), refusal rates and reasons for refusal; (2) appropriateness of data collected process and outcome measures (number of missing items and follow-up rates); (3) retention in the IDMI intervention (number of participants completed all sessions); (4) clinician competency (scores on a competency checklists); (5) feasibility of randomisation and blinding (number of refusals to be randomised and field staff’s perceptions of contamination); (6) presence of adverse advents (number of study-related adverse events reported).

The performance of the primary outcome (frequency of MA use) was measured using the timeline follow-back method (TLFB) [29]. Participants were instructed to indicate, on a printed calendar, which days in the past 2 weeks they had used MA. Although this method was originally developed to measure the frequency of alcohol use, it has been shown to be a reliable and valid method to measure illicit drug use. Self-report amphetamine use using the TLFB has resulted in both high sensitivity (0.88) and specificity (0.96) compared to urine assay results [30]. The following secondary outcomes were also measured:

*The Penn Alcohol Craving Scale (PACS) (modified for MA dependence)* is a reliable and valid five-item self-administered instrument for assessing craving for alcohol. Frequency, intensity, and duration of thoughts about MA use are assessed along with ability to resist use [31]. The PACS has shown excellent internal consistency (α = 0.92) and correlates with the Obsessive Compulsive Drinking Scale [32] and the Alcohol Urge Questionnaire [33]. The PACS has not been validated for illicit drug use. The internal consistency of the PACS in the current sample was 0.84.

*Clinical Global Impression (Severity) scale (CGI)* (Guy, 1976) is a commonly used measure of symptom severity, treatment response and the efficacy of treatments in treatment studies of patients with mental disorders. Although the tools reliability and validity has not been assessed in a substance using population it has demonstrated strong psychometric properties in different populations. For example, an investigation into the psychometric properties of the CGI among people living with schizophrenia found that the reliability of CGI was high (intraclass correlation coefficient, ICC > 0.70) and the correlation coefficients between the CGI and the Global Assessment of Functioning was high (> 0.75) [34].

*Hamilton Rating Scale for Depression (HAM-D)* (Hamilton, 1960) The HAM-D has been used internationally to assess the overall severity of depressive symptomatology and has shown reasonable validity and reliability. For example, in a systematic review of 70 published studies that used the HAM-D and found adequate internal consistency ranging from 0.46 to 0.97 and test-retest reliability ranging from 0.81 to 0.98 [35].

*Hamilton Rating Scale for Anxiety (HAM-A*) (Hamilton, 1959) The HAM-A was developed to measure the severity of anxiety symptoms. The scale consists of 14 items, each defined by a series of symptoms, and measures both psychic anxiety (mental agitation and psychological distress) and somatic anxiety (physical complaints related to anxiety). The scale is often used in randomised controlled trials (RCTs) and has shown high interrater reliability (ICC = 0.74) and concurrent validity [36].

*Sheehan Disability Scale (SDS)* was developed to assess functional impairment in three inter-related domains; work/school, social and family life. Each of the 3 response items is scored on a 10-point Likert scale [37]. A study consisting of 1001 participants found that the SDS exhibited good reliability (Cronbach’s alpha = 0.89 and inter-item correlation between 0.70 and 0.79). Further, 80% of the participants with a mental disorder showed elevated scores in this scale indicating reasonable validity [38].

## Analysis

Descriptive statistics were used to report on all feasibility outcomes. To index the relative effects of the two treatment conditions descriptive statistics were also generated for the overall sample and by treatment group. Baseline variables were tested for differences between those who completed the 3-month evaluation and those who did not using chi-square tests for categorical variables, or Fisher’s Exact where cell sizes were small, and t-tests for continuous variables, or Mann–Whitney–Wilcoxon tests if normality assumptions were not met. Linear mixed-effects regression models with repeated measures were used to assess differences in outcome measures over time between groups, with fixed effects for baseline variables that were different between groups, time, treatment group, and treatment by time interaction, and random effects for participant. The regression coefficient of interest is the treatment by time interaction. The primary regression analysis used an intention-to-treat approach with last observation carried forward (LOCF) to impute scores for missing data values. A secondary regression analysis only used data from those who completed the 3-month follow-up (no imputation). All testing was two-sided and a significance level of 0.05 was used throughout. Analyses were conducted in SAS v9.4 (SAS Institute, Inc., Cary, NC) and R v3.2.2.

## Results

A total of 278 individuals who use MA were pre-screened for possible study inclusion. From these patients, 200 (72%) were excluded. Among those who were excluded, 63 (31%) never arrived for their appointment, 30 (15%) did not have a grade nine education 7 (4%) had previously been diagnosed with a severe mental disorder. Twenty-three (11%) were receiving treatment elsewhere, 33 (16%) had not used MA in the previous 2 weeks, 25 (13%) reported that heroin was their drug of choice and 19 (10%) were living with a partner who was using.

The remaining 78 participants were assessed for study eligibility in person, where a further 18 people were excluded as they had a severe mental illness and did not report MA as their drug of choice. All participants that were eligible consented to take part and were randomly allocated. The 60 eligible patients were then randomly allocated to either the intervention or control condition. There were no refusals and concerns from participants regarding randomisation and project staff reported confidence in the randomisation and blinding process, particularly as the follow-up assessment was conducted by an individual who did not work at the hospital where the intervention sessions were being conducted. Recruitment for this feasibility trial took 9 months, approximately seven participants were recruited per month.

Table 1 shows the demographic characteristics of each group and the overall sample. There was an equal distribution of men and women in the study. The average age of participants was 31 years old (SD = 6.5), with most people initiating MA use at 21 years of age (SD = 5.9). Most participants were single (n = 53; 88.3%) and unemployed (n = 47; 78.3%). More Coloured participants (88.3%) participated in the intervention than Black (8.3%) or White (3.3%). These categories are not intended to reify sociocultural constructs, but rather to ensure that ongoing health disparities across groups can be explored.

**Table 1:** Baseline characteristics of participants, n = 60

Variable	Total sample (n = 60)	Control (n = 30)	IDMI (n = 3 0)	p-value
Age (mean, sd)	31.0 (6.5)	30.8 (6.7)	31.1 (6.4)	0.99
Gender (n, %)				
Male	32 (53.3)	13 (43.3)	19 (63.3)	0.12
Female	28 (46.7)	17 (56.7)	11 (36.7)	
Race (n, %)				
Black	5 (8.3)	4 (13.3)	1 (3.3)	0.51
Coloured	53 (88.3)	25 (83.3)	28 (93.3)	
White	2 (3.3)	1 (3.3)	1 (3.3)	
Relationship status (n, %)				
Single	53 (88.3)	27 (90.0)	26 (86.7)	0.99
In relationship	7 (11.7)	3 (10.0)	4 (13.3)	
Education (n, %)				
Did not finish high school	29 (48.3)	10 (33.3)	19 (63.3)	0.02*
Finished high school	31 (51.7)	20 (66.7)	11 (36.7)	
Employment (n, %)				
Unemployed	47 (78.3)	24 (80.0)	23 (76.7)	0.75
Employed	13 (21.7)	6 (20.0)	7 (23.3)	
Age at first use (mean, sd)	21.0 (5.9)	21.0 (6.0)	21.1 (5.8)	0.85
Received previous treatment (n, %)	28 (46.7)	12 (40.0)	16 (53.3)	0.30

Of the 30 participants who were randomised to the intervention group, 26 completed session one, 24 completed two sessions, 21 completed three sessions, 18 completed four sessions, 15 completed five sessions and 13 (43%) completed all six sessions. The reasons provided for treatment suspension included: imprisonment (n = 3), employment (n = 5) and loss of communication (n = 9). For the control group, 10 of the 30 participants attended at least one session at the outpatient facility, with a maximum of 3 sessions they were referred to. Three of the 30 participants were admitted to an inpatient treatment centre.

In total, 39 (65%) participants completed the 6-week follow-up and 40 (67%) completed the 3-month follow-up. Age at time of first use and age at baseline were the only variables that distinguished between participants who completed the 3-month follow-up and those who did not (age at first use: 22.3 years vs. 18.6 years, p = 0.04; age at baseline: 32.2 years vs. 28.6 years, p = 0.06). There were no significant differences between groups in terms of follow-up rates at 6-weeks and 3 months. See Table 2.

**Table 2:** Means and standard deviations of groups at baseline, 6 weeks and 3 months follow-up

Variable	Control (mean [sd])	IDMI (mean [sd])
Substance use (days in 2 weeks)		
Baseline	8.57 (4.29)	9.73 (3.95)
6 week follow-up	6.43 (4.62)	4.83 (4.86)
3 month follow-up	6. 90 (4.12)	4.80 (4.97)
Cravings (PENN craving)		
Baseline	22.50 (6.85)	20.37 (7.50)
6 week follow-up	17.43 (8.35)	15.97 (7.61)
3 month follow-up	13.13 (10.09)	14.83 (8.35)
Depression (HAM-D)		
Baseline	12.50 (5.25)	12.90 (6.22)
6 week follow-up	11.23 (5.83)	10.20 (6.50)
3 month follow-up	8.40 (5.37)	8.53 (6.69)
Anxiety (HAM-A)		
Baseline	20.27 (10.19)	18.70 (10.02)
6 week follow-up	17.73 (10.08)	13.87 (9.27)
3 month follow-up	14.63 (9.44)	8.79 (8.00)
Clinical Global Impressions Scale (CGI)		
Baseline	5.13 (0.97)	5.27 (0.91)
6 week follow-up	4.57 (1.72)	4.03 (1.65)
3 month follow-up	4.00 (1.89)	3.60 (1.81)
Sheehan disability (family)		
Baseline	8.40 (2.43)	7.77 (2.91)
6 week follow-up	6.47 (4.02)	5.53 (3.69)
3 month follow-up	4.50 (3.97)	4.43 (3.31)
Sheehan disability (work)		
Baseline	6.07 (3.85)	7.30 (2.49)
6 week follow-up	4.70 (4.27)	5.50 (3.42)
3 month follow-up	4.00 (3.80)	4.50 (3.75)
Sheehan disability (social life)		
Baseline	7.78 (2.27)	7.50 (2.86)
6 week follow-up	5.53 (4.08)	4.70 (3.96)
3 month follow-up	3.63 (3.93)	4.27 (3.63)

**Fig. 3:**
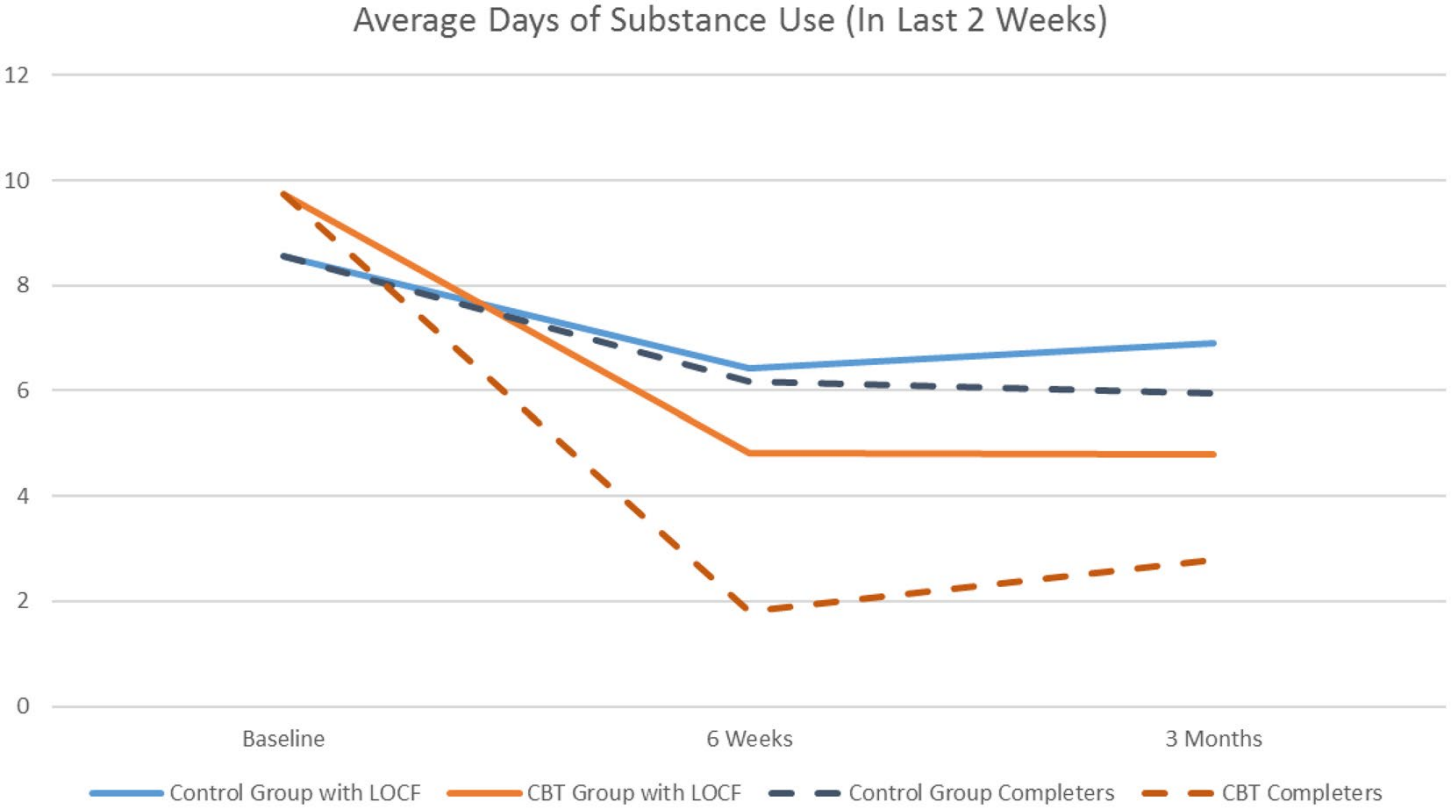
Graph of average days of substance use over time by group

Improvements on all outcomes over time were observed for both the intervention group and the control group (see Table 3). In the intention-to-treat analysis, the IDMI group displayed greater reductions in frequency of MA use (the primary outcome) than the control group. Results of the linear mixed-effects regression models revealed a statistically significant time by treatment group interaction (p = 0.01) at both 6 weeks (r = − 4.78, p < 0.01) and 3 months (r = − 4.63, p < 0.01) (Table 3). This finding was consistent when looking at the respondents who completed the 3-month follow-up only (p = 0.002) at 6 weeks (r = − 4.78, p < 0.01) and 3 months post-intervention (r = − 4.63, p < 0.01) (Table 4). Figure 3 shows the average number of days using MA in the previous 2 weeks over time for the intention to treat (ITT) analysis with LOCF and the analysis with study completers only.

**Table 3:** Intention to Treat Comparison of Groups on Primary and Secondary Outcomes

	Dependent variable							
	Days used	Penn total	SD work	SD social life	SD family	CGI	HAM-A	HAM-D
*Time period (reference: baseline; type III SS p-value)*	*0.029*	*< 0.001*	*0.023*	< *0.001*	< *0.001*	*0.002*	*0.001*	<* 0.001*
6 week follow-up	− 2.133** (0.830)	− 5.067*** (1.523)	− 1.367* (0.751)	− 2.233*** (0.755)	− 1.933*** (0.697)	− 0.567* (0.312)	− 2.533* (1.412)	− 1.267 (0.952)
3 month follow-up	− 1.667** (0.830)	− 9.367*** (1.523)	− 2.067*** (0.751)	− 4.133*** (0.755)	− 3.900*** (0.697)	− 1.133*** (0.312)	− 5.633*** (1.412)	− 4.100*** (0.952)
*Treatment group (reference: control; type III SS p-value)*	*0.343*	*0.347*	*0.233*	*0.791*	*0.474*	*0.915*	*0.319*	*0.899*
Intervention	1.104 (1.155)	− 2.020 (2.131)	1.129 (0.937)	− 0.242 (0.909)	− 0.645 (0.894)	0.041 (0.387)	− 2.302 (2.458)	0.230 (1.575)
*Age at baseline (type III SS p-value)*	*0.337*	*0.279*	*0.106*	*0.072*	*0.156*	*0.093*	*0.497*	0.499
	− 0.070 (0.072)	− 0.145 (0.133)	− 0.089 (0.054)	− 0.094* (0.051)	− 0.076 (0.053)	− 0.038* (0.022)	0.087 (0.167)	0.052 (0.105)
*CGI-severity at baseline (type III SS p-value)*	*0.197*	*0.185*	*0.826*	*0.449*	*0.466*	*N/A*	*0.006*	*0.002*
	0.660 (0.505)	− 1.391 (1.037)	− 0.094 (0.424)	0.305 (0.400)	0.303 (0.413)		3.489*** (1.225)	2.435*** (0.760)
*Number of days in past 2 weeks using meth at baseline (type III SS p-value)*	*N/A*	*0.651*	*0.191*	*0.774*	*0.992*	*0.013*	*0.381*	*0.496*
		0.107 (0.236)	0.128 (0.097)	− 0.026 (0.091)	− 0.001 (0.094)	0.091** (0.036)	0.602** (0.265)	0.129 (0.167)
*Time period by treatment group interaction (reference: baseline control; type III SS p-value)*	*0.013*	0.169	*0.786*	*0.386*	*0.672*	*0.283*	*0.312*	*0.529*
6 week follow-up Intervention	− 2.767** (1.173)	0.667 (2.154)	− 0.433 (1.063)	− 0.567 (1.068)	− 0.300 (0.985)	− 0.667 (0.442)	− 2.300 (1.997)	− 1.433 (1.346)
3 month follow-up Intervention	− 3.267*** (1.173)	3.833* (2.154)	− 0.733 (1.063)	0.900 (1.068)	0.567 (0.985)	− 0.533 (0.442)	− 2.900 (1.997)	− 0.267 (1.346)
*Constant*	7.336** (3.618)	33.193*** (6.648)	8.201*** (2.730)	9.324*** (2.580)	9.194*** (2.655)	5.526*** (0.812)	12.418** (5.975)	9.780** (3.774)
Observations	180	180	180	180	180	180	180	180
Log Likelihood	− 499.304	− 603.844	− 472.698	− 470.088	− 462.489	− 321.576	− 609.198	− 537.667
Akaike Inf. Crit.	1,018.608	1,229.687	967.396	962.175	946.978	663.153	1,238.396	1,095.333
Bayesian Inf. Crit.	1050.083	1264.245	1001.955	996.734	981.537	694.628	1269.870	1126.808

Linear mixed-effects model; p-values entered in italic text; all other values are coefficients (standard error) unless otherwise stated

*p < 0.1; **p < 0.05; ***p < 0.01

**Table 4:** Comparison of groups on primary and secondary outcomes at 3-month follow-up: completers only

	Dependent variable							
	Days used	Penn total	SD work	SD Social life	SD family	CGI	HAM-A	HAM-D
*Time period (reference: baseline; type III SS p-value)*	*0.007*	< *0.001*	*0.011*	< *0.001*	< *0.001*	*0.001*	*0.002*	< *0.001*
6 week follow-up	− 3.223*** (1.043)	− 9.070*** (1.908)	− 2.590** (1.050)	− 4.060*** (1.009)	− 3.383*** (0.978)	− 1.013** (0.415)	− 3.332* (1.953)	− 2.444* (1.333)
3 month follow-up	− 2.318** (0.982)	− 12.545*** (1.785)	− 2.761*** (0.982)	− 5.580*** (0.948)	− 5.322*** (0.912)	− 1.631*** (0.396)	− 6.770*** (1.850)	− 6.073*** (1.288)
*Treatment group (reference: control; type III SS p-value)*	*0.256*	*0.297*	*0.152*	*0.990*	*0.918*	*0.900*	*0.720*	*0.856*
Intervention	1.342 (1.163)	− 2.533 (2.392)	1.652 (1.128)	− 0.013 1.015)	− 0.114 (1.106)	− 0.060 (0.456)	− 1.009 (2.792)	0.305 (1.666)
*Age at baseline (type III SS p-value)*	*0.866*	*0.899*	*0.834*	*0.972*	*0.527*	*0.874*	*0.105*	*0.021*
	0.010 (0.056)	0.016 (0.126)	− 0.011(0.052)	− 0.002 (0.043)	− 0.035 (0.055)	− 0.003 (0.021)	0.265 (0.159)	0.212** (0.088)
*CGI-severity at baseline (type III SS p-value)*	*0.060*	*0.455*	*0.321*	*0.418*	*0.924*	*N/A*	*0.067*	*0.139*
	0.924* (0.476)	− 1.002 (1.325)	− 0.564 (0.560)	− 0.377 (0.459)	− 0.056 (0.581)		3.163* (1.671)	1.411 (0.932)
*Number of days in past 2 weeks using meth at baseline (ttype III SS p-value)*	*N/A*	*0.991*	*0.173*	0.050	*0.138*	*0.009*	*0.564*	*0.925*
		− 0.003 (0.280)	0.167 (0.120)	0.201** (0.099)	0.188 (0.124)	0.107*** (0.039)	0.205 (0.353)	− 0.019 (0.198)
*Time period by treatment group interaction (reference: baseline control; type III SS p-value)*	*0.002*	*0.230*	*0.531*	*0.753*	*0.948*	*0.171*	*0.049*	*0.660*
6 week follow-up Intervention	− 4.783*** (1.550)	2.041 (2.833)	− 0.603 (1.543)	− 0.249 (1.483)	0.128 (1.436)	− 1.123* (0.624)	− 5.460* (2.937)	− 1.817 (2.005)
3 month follow-up Intervention	− 4.626*** (1.463)	4.601* (2.661)	− 1.628 (1.453)	0.802 (1.403)	0.433 (1.349)	− 0.813 (0.586)	− 6.452** (2.732)	− 0.594 (1.886)
*Constant*	3.232 (3.134)	27.087*** (7.127)	7.266** (2.999)	7.905*** (2.466)	8.033** (3.103)	4.353*** (0.815)	− 6.052 (8.957)	(4.983) − 1.249
Observations	113	113	111	111	111	111	111	110
Log Likelihood	− 297.348	− 367.367	− 289.208	− 280.067	− 285.397	− 196.906	− 369.688	− 320.790
Akaike Inf. Crit.	614.695	756.734	600.417	582.133	592.794	413.812	761.376	663.580
Bayesian Inf. Crit.	641.235	785.822	629.292	611.008	621.668	440.160	790.251	692.346

Linear mixed-effects model; p-values entered in italic text; all other values are coefficients (standard error) unless otherwise stated

*p < 0.1; **p < 0.05; ***p < 0.01

In addition, analysis of secondary measures demonstrated a significant difference in the HAM-A score between the groups in the analysis that included completers only (Table 4). The intervention group reported significantly lower scores on the HAM-A than the control group (p = 0.049) at both 6 weeks and at 3 months post-intervention. There were no significant differences between groups for the other secondary outcome measures.

## Discussion

This study is among the first to examine the feasibility of a brief treatment using imaginal desensitisation plus motivational interviewing for MA dependence in a low- and middle-income country context. Findings suggest that although it is feasible to successfully identify and recruit participants to participate in an intervention delivered by master’s level clinical psychologists, retention and follow-up rates could be improved. Although preliminary results indicate that this intervention may be an efficacious intervention for reducing risk for MA, a number of modifications to the study design will need to be considered when progressing towards a future definitive randomised controlled trial. Findings indicate that it is feasible to identify and recruit individuals who use MA for such an intervention programme and that it is urgently needed among this population. Of the 278 MA users that contacted the research team, over half had never accessed substance use treatment services previously, and those who had were seeking treatment alternatives. In a recent systematic review investigating barriers for MA users to access treatment, the most common barriers highlighted were attitudinal in nature. These included the belief that treatment was not needed, stigma, a preference to be on their own to withdraw, and privacy and confidentiality issues [39]. Many of these barriers may have been reduced in the present study where an individualized, brief treatment was provided outside of known specialist substance use treatment settings.

Nevertheless, keeping patients with a substance use disorder actively engaged in treatment posed many challenges. In this study, only 43% of participants in the intervention arm completed all six sessions. Consistent with the data here, an evaluation of the implementation of the Matrix model at one clinic in the Western Cape found that over six years of the 2,233 clients who completed screening, approximately 44% (n = 986) initiated treatment. Among those who initiated treatment, 45% completed at least four group sessions, 30% early recovery skills training (minimum eight group sessions), and 13% completed the full Matrix programme [22]. Other studies have also demonstrated high dropout rates from outpatient services in this province, particularly in the first 2 weeks of treatment [40].

Identifying methods to optimise treatment retention is crucial when considering the implementation of a definitive randomized control trial. To address this concern, we are considering using mobile technology to deliver the intervention. Given the availability of smart phones in this context, technology-assisted interventions may provide a viable alternative to some of the face-to-face treatments [41]. In our experience, it is feasible to follow-up with substance using populations with much lower attrition rates should sufficient funds be available to track participants and conduct follow-ups in the community [42].

Second, results from this feasibility trial provide guidance on potentially useful outcome measures and provide some indications of preliminary effect sizes that could help sample size calculations for a larger, more definitive trial. Despite limitations, results also suggest that this intervention may be an efficacious intervention for reducing frequency of MA use. In this feasibility RCT, the intervention significantly reduced frequency of MA use in comparison to usual care. Participants in the intervention group reported significantly fewer days of MA use compared to the control group at both the 6 week and 3-month follow-up assessments. These findings are consistent with the available literature on the effectiveness of CBT-based approaches [14], where for example small to medium reductions are reported [43].

However, the intervention effect appears to diminish over time. This drop off is not surprising given evidence that after 90 day of treatment MA users are particularly vulnerable for relapse. It has been argued that this period is associated with anhedonia that arises as neurotransmitter systems recover after extended periods of MA use [44]. Furthermore, other studies investigating the efficacy of psychosocial treatments for MA use also suggest that treatment effects decline over time [14, 45]. Given this evidence, additional efforts to improve the duration of the intervention effect, such as additional intervention sessions, are warranted when considering a future definitive trial.

While our findings provide preliminary evidence that a six-session brief treatment holds promise for facilitating changes in MA use, in addition to efforts aimed at retention and intervention effect, further amendments to the study design will need to be considered when progressing towards a future definitive trial. First, participants in the control condition received a referral to existing specialist outpatient facilities where variations in treatments and dosage were reported for the very limited number of participants who accessed any treatment. The lack of uniformity in the treatment provided to the control group could influence the internal validity of the study potentially compromising the results. Further consideration on the most appropriate comparison group is essential when taking this to a definitive trial. Second, relying on self-reports of MA use as an outcome is problematic. Consideration must be given to the use of sophisticated biological markers of MA use (such as hair sample testing) that allow for assessments of the levels of MA use (and not just the presence or absence of metabolites) is warranted given the harm reduction rather than abstinence focus of the intervention. Third, grocery vouchers were given to participants at each assessment and treatment session and transport costs to attend intervention sessions were covered. This could have potentially influenced participants motivation to participate in the study and could have contributed to the better retention rates in the intervention group (as the control group were not incentivised to attend treatment). Given that this type of incentive is unlikely to be provided in a real-world setting, it is recommended that these incentives are not provided in a definitive trial. Finally, this intervention was delivered by master’s level psychologists, of which there are few in the public service. Future studies should explore whether this brief treatment could be task-shared to registered counsellors and community health workers, given that it is both structured and manualised. In South Africa, there is a small but growing body of literature to show that with training and ongoing supervision, community health workers are able to deliver a range of evidence-based psychological treatments [46].

We also acknowledge that an intervention, such as the one described, cannot be considered a “solution” in isolation. It does not address the systemic issues within which substance use disorders arise, such as high levels of unemployment and social inequality, which may have a maintaining effect on continued problematic use [47]. Adequately addressing problematic MA use requires intervention beyond the health care and social service sectors.

## Conclusions

The results of the study suggest that it is feasible to successfully identify and recruit participants to participate in a brief IDMI treatment for MA use delivered by master’s level clinical psychologists. Our findings provide preliminary evidence that a six-session brief treatment holds promise for facilitating changes in MA use should concerns around retention and intervention effect be addressed. Findings provide evidence to support progression to a larger more definitive trial, pending proposed changes to the recruitment, intervention and retention protocols.

## Data Availability

The dataset is available from the corresponding author on reasonable request.

## Abbreviations

MAUD: Methamphetamine use disorders
IDMI: Imaginal desensitisation plus motivational interviewing
MA: Methamphetamine
HIV: Human Immunodeficiency Virus
CBT: Cognitive behaviour therapy
CONSORT: Consolidated Standards of Reporting Trials
SPIRIT: Recommendations for Interventional Trials
NGOs: Non government Organisations
DSM-IV: Diagnostic and Statistical Manual fourth edition
MINI: Mini-International Psychiatric Interview
TLFB: Timeline follow-back method
PACS: Penn Alcohol Craving Scale
CGI: Clinical Global Impression (Severity) scale
HAM-D: Hamilton Rating Scale for Depression
HAM-A: Hamilton Rating Scale for Anxiety
RCTs: Randomised controlled trials
SDS: Sheehan Disability Scale
ITT: Intention to treat
LOCF: Last observation carried forward

## References

[1] Forouzanfar MH, Alexander  L, Anderson HR, Bachman VF, Biryukov S, Brauer M (2015). Global, regional, and national comparative risk assessment of 79 behavioural, environmental and occupational, and metabolic risks or clusters of risks in 188 countries, 1990–2013: a systematic analysis for the Global Burden of Disease Study 2013. Lancet (London, England).

[2] Stein DJ, Seedat S, Herman A, Moomal H, Heeringa SG, Kessler RC (2008). Lifetime prevalence of psychiatric disorders in South Africa. Br J Psychiatry J Ment Sci.

[3] Pluddemann A, Myers BJ, Parry CD (2008). Surge in treatment admissions related to methamphetamine use in Cape Town, South Africa: implications for public health. Drug Alcohol Rev.

[4] Peltzer K, Simbayi L, Kalichman S, Jooste S, Cloete A, Mbelle M (2009). Drug use and HIV risk behaviour in three urban South African communities. J Soc Sci.

[5] Meredith CW, Jaffe C, Ang-Lee K, Saxon AJ (2005). Implications of chronic methamphetamine use: a literature review. Harv Rev Psychiatry.

[6] Darke S, Kaye S, McKetin R, Duflou J (2008). Major physical and psychological harms of methamphetamine use. Drug Alcohol Rev.

[7] Kaye S, McKetin R, Duflou J, Darke S (2007). Methamphetamine and cardiovascular pathology: a review of the evidence. Addiction.

[8] Watt MH, Myers B, Towe SL, Meade CS (2015). The mental health experiences and needs of methamphetamine users in Cape Town: a mixed methods study. S Afr Med J Suid-Afrikaanse tydskrif vir geneeskunde.

[9] Meade CS, Towe SL, Watt MH, Hobkirk AL, Skinner D, Myers B (2015). HIV testing behaviors and attitudes among community recruited methamphetamine users in a South African township. AIDS Behav.

[10] Pluddemann A, Flisher AJ, McKetin R, Parry C, Lombard C (2010). Methamphetamine use, aggressive behavior and other mental health issues among high-school students in Cape Town, South Africa. Drug Alcohol Depend.

[11] Stuart AM, Baker AL, Denham AMJ, Lee NK, Hall A, Oldmeadow C (2020). Psychological treatment for methamphetamine use and associated psychiatric symptom outcomes: A systematic review. J Subst Abuse Treat.

[12] Minozzi S, Saulle R, De Crescenzo F, Amato L (2016). Psychosocial interventions for psychostimulant misuse. Cochrane Database Syst Rev.

[13] Shoptaw SJ, Kao U, Heinzerling K, Ling W (2009). Treatment for amphetamine withdrawal. Cochrane Database Syst Rev.

[14] Lee NK, Rawson RA (2008). A systematic review of cognitive and behavioural therapies for methamphetamine dependence. Drug Alcohol Rev.

[15] Degenhardt L, Glantz M, Evans-Lacko S, Sadikova E, Sampson N, Thornicroft G (2017). Estimating treatment coverage for people with substance use disorders: an analysis of data from the World Mental Health Surveys. World Psychiatry.

[16] Meade CS, Towe SL, Watt MH, Lion RR, Myers B, Skinner D (2015). Addiction and treatment experiences among active methamphetamine users recruited from a township community in Cape Town, South Africa: a mixed-methods study. Drug Alcohol Depend.

[17] Myers B, Kline TL, Doherty IA, Carney T, Wechsberg WM (2014). Perceived need for substance use treatment among young women from disadvantaged communities in Cape Town, South Africa. BMC Psychiatry.

[18] Myers B, Fakier N (2008). Alcohol and drug abuse: removing structural barriers to treatment for historically disadvantaged communities in Cape Town. Int J Soc Welfare.

[19] Myers B, Louw J, Pasche S (2015). Inequitable access to substance abuse treatment services in Cape Town, South Africa. Substance Abuse Treat Prevent Policy.

[20] Rawson RA, Shoptaw SJ, Obert JL, McCann MJ, Hasson AL, Marinelli-Casey PJ (1995). An intensive outpatient approach for cocaine abuse treatment. The Matrix model. J Substance Abuse Treat.

[21] Shoptaw S, Rawson RA, McCann MJ, Obert JL (1994). The Matrix model of outpatient stimulant abuse treatment: evidence of efficacy. J Addictive Dis.

[22] Gouse H, Magidson JF, Burnhams W, Remmert JE, Myers B, Joska JA (2016). Implementation of cognitive-behavioral substance abuse treatment in Sub-Saharan Africa: treatment engagement and abstinence at treatment exit. PLoS ONE.

[23] Grant JEDC, Odlaug BL (2011). Treatments that work: treating impulse control disorders: a cognitive-behavioral therapy program, therapist guide.

[24] Grant JE, Donahue CB, Odlaug BL, Kim SW, Miller MJ, Petry NM (2009). Imaginal desensitisation plus motivational interviewing for pathological gambling: randomised controlled trial. Br J Psychiatry J Ment Sci.

[25] Pasche SC, Sinclair H, Collins P, Pretorius A, Grant JE, Stein DJ (2013). The effectiveness of a cognitive-behavioral intervention for pathological gambling: a country-wide study. Ann Clin Psychiatry.

[26] Grant JE, Potenza MN, Weinstein A, Gorelick DA (2010). Introduction to behavioral addictions. Am J Drug Alcohol Abus.

[27] Eldridge SM, Chan CL, Campbell MJ, Bond CM, Hopewell S, Thabane L (2016). CONSORT 2010 statement: extension to randomised pilot and feasibility trials. Pilot Feasibil Stud.

[28] Chan AW, Tetzlaff JM, Altman DG, Laupacis A, Gotzsche PC, Krle AJK (2015). SPIRIT 2013 Statement: defining standard protocol items for clinical trials. Rev Panam Salud Publica.

[29] Sobell LC, Maisto SA, Sobell MB, Cooper AM (1979). Reliability of alcohol abusers’ self-reports of drinking behavior. Behav Res Ther.

[30] Fals-Stewart W, O’Farrell TJ, Freitas TT, McFarlin SK, Rutigliano P (2000). The timeline followback reports of psychoactive substance use by drug-abusing patients: psychometric properties. J Consult Clin Psychol.

[31] Flannery BA, Volpicelli JR, Pettinati HM (1999). Psychometric properties of the Penn Alcohol Craving Scale. Alcohol Clin Exp Res.

[32] Anton RF, Moak DH, Latham P (1995). The Obsessive Compulsive Drinking Scale: a self-rated instrument for the quantification of thoughts about alcohol and drinking behavior. Alcohol Clin Exp Res.

[33] Bohn MJ, Krahn DD, Staehler BA (1995). Development and initial validation of a measure of drinking urges in abstinent alcoholics. Alcohol Clin Exp Res.

[34] Haro JM, Kamath SA, Ochoa S, Novick D, Rele K, Fargas A (2003). The Clinical Global Impression-Schizophrenia scale: a simple instrument to measure the diversity of symptoms present in schizophrenia. Acta Psychiatrica Scand Supplement.

[35] Bagby RM, Ryder AG, Schuller DR, Marshall MB (2004). The Hamilton Depression Rating Scale: has the gold standard become a lead weight?. Am J Psychiatry.

[36] Maier W, Buller R, Philipp M, Heuser I (1988). The Hamilton Anxiety Scale: reliability, validity and sensitivity to change in anxiety and depressive disorders. J Affect Disord.

[37] Sheehan KH, Sheehan DV (2008). Assessing treatment effects in clinical trials with the discan metric of the Sheehan Disability Scale. Int Clin Psychopharmacol.

[38] Leon AC, Olfson M, Portera L, Farber L, Sheehan DV (1997). Assessing psychiatric impairment in primary care with the Sheehan Disability Scale. Int J Psychiatry Med.

[39] Cumming C, Troeung L, Young JT, Kelty E, Preen DB (2016). Barriers to accessing methamphetamine treatment: a systematic review and meta-analysis. Drug Alcohol Depend.

[40] Myers B, Williams PP, Govender R, Manderscheid R, Koch JR (2018). Substance abuse treatment engagement, completion and short-term outcomes in the Western Cape province, South Africa: Findings from the Service Quality Measures Initiative. Drug Alcohol Depend.

[41] Keoleian V, Stalcup SA, Polcin DL, Brown M, Galloway G (2013). A cognitive behavioral therapy-based text messaging intervention for methamphetamine dependence. J Psychoactive Drugs.

[42] Wechsberg WM, Jewkes R, Novak SP, Kline T, Myers B, Browne FA (2013). A brief intervention for drug use, sexual risk behaviours and violence prevention with vulnerable women in South Africa: a randomised trial of the Women’s Health CoOp. BMJ Open.

[43] Baker A, Boggs TG, Lewin TJ (2001). Randomized controlled trial of brief cognitive-behavioural interventions among regular users of amphetamine. Addiction.

[44] O’Neill J, Tobias MC, Hudkins M, London ED (2014). Glutamatergic neurometabolites during early abstinence from chronic methamphetamine abuse. Int J Neuropsychopharmacol.

[45] Brecht ML, Herbeck D (2014). Time to relapse following treatment for methamphetamine use: a long-term perspective on patterns and predictors. Drug Alcohol Depend.

[46] Spedding MF, Stein DJ, Sorsdahl KR (2015). Task-shifting psychosocial interventions in public mental health: a review of the evidence in the South African context. South Afr Health Rev.

[47] Hobkirk AL, Watt MH, Myers B, Skinner D, Meade CS (2016). A qualitative study of methamphetamine initiation in Cape Town, South Africa. Int J Drug Policy.

